# Atlanta Academy of Medicine

**Published:** 1876-05

**Authors:** R. W. Westmoreland

**Affiliations:** Reporter


### Atlanta Academy of Medicine.

R. W. WESTMORELAND, M.D., REPORTER.

ATLANTA, March 7, 1876.

Meeting was called to order; the President, Dr. Miller, in the chair.

Dr. Armstrong referred to the report of an operation for piles, and remarked that he reported no hemorrhage. He understood afterward, that as the result of a purgative, a good deal of clotted blood was discharged.

Dr. Taliaferro reported a case of labor, in which, at the earnest entreaties of the woman, chloroform was administered by inhalation, in small doses. When the patient was somewhat under the influence of the medicine, the pains slackened up, but upon its withdrawal they recurred in full force. This alternative was continued until he was satisfied that the chloroform caused the sedative effect upon the uterus. After the birth of the child, severe hemorrhage took place, accompanied by rigors and faintness. The hemorrhage he attempted to check by introducing his hand into the uterus and manipulating in conjunction with his other hand on the outside, but produced no contraction and control of hemorrhage. Finally, by the hand acting as a tampon, and the administration of ergot, he produced contraction of the uterus, and consequent stoppage of bleeding. Remarked that he considered this as a case of great interest, as it was an undecided question as to the uterine sedative effect of chloroform; he having believed heretofore that it was not.

Dr. Todd inquired of Dr. Taliaferro his experience with the fluid extract of ergot and of ergotine—whether favorable.

Dr. Taliaferro replied that it was not very favorable.

Dr. W. F. Westmoreland stated that of all the preparations of ergot, he thought the wine was preferable. He felt satisfied that the contraction produced by this preparation had produced death of the fœtus in two cases that he knew of. Given in the dose of from flʒ to a fl℥, according to circumstances, its full effect would be experienced. Remarked that he would like to hear expressions of opinion upon the subject from members.

Dr. Taliaferro mentioned the supposed antagonistic qualities of opium and ergot, and inquired if the experience of members supported that idea.

Dr. Baird remarked that he had used ergotine by hypodermic injection, in congestion of spinal cord and brain, with very marked and undeniable benefit. Usual dose, one grain. Has used fluid extract in from one-half to one fl3 doses. Reported a case in which the hypodermic injection was used in congestion of brain, with cure. Remarked that this was, as he thought, a correllary effect to that had on the uterus, by exercising the involuntary muscles.

Dr. W. F. Westmoreland remarked that Dr. Baird's idea was in conformity with the general idea of the day, that local applications of ergot had local effects, and general applications general effects. He does not believe in the local application having local effect alone.

Dr. Baird, on inquiry by Dr. John M. Johnson as to the quantity necessary as a parturient, replied that his experience would not justify a suggestion, but he was inclined to favor the fluid extract in about one fl3 doses.

Dr. Boring being called on for his experience in the use of ergot, replied that his usual practice was to leave nature to herself. Was of the opinion that physicians are often too anxious to interfere, and do injury where patience would accomplish much good. He said he had seen cases where ergot would produce contractions very readily; then again others in which no visible effect could be produced. His usual occasion for administering ergot is in hemorrhagic cases. For its antihemorrhagic effect, gives it in the doses of fl3j, three or four times a day.

Upon inquiry by Dr. Taliaferro, if he did not consider it a uterine stimulant, he replied that he did not consider that it failed at all times to produce contraction, but that it often did.

Dr. Taliaferro thought that the uncertainty in the use of ergot was occasioned by the impurity of the drug. Especially was this true, in his opinion, with the fluid extract. Thought the pure drug one of the few specifics in the materia medica.

Dr. Connally remarked that he relied on the decoction. Desired to revert to Dr. Taliaferro's case as corroborative of an article he had lately read in regard to chloroform being a frequent cause of post-partum hemorrhage. This article, he remarked, had mentioned the fact that the chloroform smell had been discovored in the breath of the fœtus. Coincided with Dr. Baird in believing ergot beneficial in congestion of brain. He usually gave it in conjunction with bromide potassium.

Dr. Miller coincided with Drs. Boring and W. F. Westmoreland. Believes that ergot undoubtedly produces contraction of the uterus, and has this effect through the spinal cord. Denied the instance of muscular tissue in the blood-vessels, and therefore could not agree with Dr. Baird, that congestion was controlled with ergot, by contraction of this supposed tissue. Remarked that he never heard of the fact that chloroform produced post-partum hemorrhage or injury to the fœtus, but believed, with Dr. Taliaferro, that it interfered with the pains.

Dr. Todd disagreed with Dr. Miller as to the existence of muscular tissue in the blood-vessels, and coincided with Dr. Baird in the *modus operandi* of the effect of ergot in congestion. Thought, also, that ergot, in over-doses, produced delirium.

Dr. W. F. Westmoreland, in corroboration of the fact that the medicine acted upon the blood-vessels by contraction, mentioned the existence of dry gangrene of the toes in some parts of France, from eating spurred rye.

Dr. John M. Johnson discussed the merits and demerits of the remedy at some length, and expressed his preference for the decoction of ergot, though he often used the wine, because he could not take time to prepare the decoction. Altogether, does not have much faith in the remedy, and is totally opposed to all the opinions advanced in favor of it.

Discussion was prolonged by Drs. Todd, W. F. Westmoreland, Miller, and Baird.

Dr. Calhoun reported the case of a young lady, aged 20, affected with cystic tumor in the external canthus of right eye, extending back as far as the optic nerve, between the bone and ball of eye, producing an effect on muscles of lid called

*ptosis.* No fluctuation could be discovered. An eliptical incision was made, one inch in length, and the tumor was dissected out carefully. The tumor was found to be flattened, and extending, between the inferior and external recti muscles, quite to the optic nerve. In the course of dissecting out the tumor, a small puncture was made into the capsule, followed by the escape of a watery liquid. This growth had very much the appearance of a fatty tumor, on account of the deposition of the fatty substance around the capsule. Upon being satisfied that all the tumor had been removed, the wound was brought together by silver sutures. Considerable ecchymosis resulted under the conjunctiva, which, however, readily disappeared on the use of cold applications. The lower lid droops a little, but is fast recovering its normal condition. The peculiarity of the case was the extreme extent and size of a tumor of this description and in this locality.

(The condition of ptosis has entirely disappeared since the above report.—Reporter.)

Academy adjourned.

---

Atlanta, March 14, 1876.

Meeting called to order by the President, Dr. Miller.

Dr. W. F. Westmoreland reported the occurrence of extentensive hemorrhage in operating for hemorrhoids with the ecraseur. Remarked that it was the first time he had ever had such result from operations with this instrument.

The discussion of last meeting, in reference to the existence of muscular tissue in the blood vessels, was resumed by Drs. Miller and Baird, quoting from various authorities, corroborative each of his doctrine. Members expressed their opinions on the subject, and finally agreed that much doubt existed either one way or the other.

On motion, academy adjourned.

---

H. F. SCOTT, M.D., Reporter.

---

Atlanta, March 21, 1876.

Academy met, Dr. Miller, President, presiding.

Dr. Armstrong wished to call attention to the case of an old gentleman, which had given him some trouble, and desired an expression of opinion from the Academy. In this case, hemiplegia suddenly occurred, with slight paralysis of the muscles of the face. The mind, however, remained clear throughout, and all the functions perfect, except a disturbance about the bladder. He has suffered from cystitis for years, with an occasional trouble in emptying his bladder. His appetite and digestion have remained good. On examination, he found, as the cause of the cystitis, enlargement of the prostate gland, with probably some disturbance about the kidneys. His only treatment has been constantly drawing off the urine and washing out the bladder once or twice daily with warm water; suppositories of opium to relieve and secure rest.

In reply to question, said he had used only warm water to wash out the bladder; he has used in some cases carbolic acid, Labarraque's Solution, and like remedies of proper strength, but not in this case.

Dr. Connally mentioned that one or two cases had been reported by Drs. Logan and Willis F. Westmoreland, to the Academy, of the use of ergot in enlargement of the prostate gland, and inquired if it had been used in the above case.

Dr. Armstrong, in reply, said he had given no ergot in this case; he has, however, given this remedy in some cases, but with no decided and permanent improvement. He remarked further, that the history of such cases was that they were paroxysmal, being better at times, and again worse; hence it was difficult to judge of the value of any particular remedy.

Dr. Calhoun knew of surgeons who rely on warm water altogether in the treatment of cystitis. They wash out the bladder once or twice daily with it, putting in sometimes a little salt. He called attention to the fact that Prof. Simon, of Heidelberg, had originated the plan of dilating the uterus for certain kidney and bladder affections in the female. Prof. Simon was the only man who had ever successfully removed a kidney. He also dilated the rectum, and by inserting his hand and pushing the intestine up until he brought his fingers to the under surface of the liver, was enabled to detect any irregularities about that viscus.

In reply to a question from the President, as to the expediency of using nitrate of silver in cystitis, Dr. John G. Westmoreland called to mind two cases in his practice in which he used it in the strength of from eight to fifteen grains to the ounce, and after several applications they were cured. In acute cystitis he prefers the sulphate of cadmium; whilst in chronic cases he would use the silver. In neither of his cases was there any enlargement of prostate gland, so far as was known.

The President called attention to the fact that in inflammations of all other mucous membranes silver is used, and he knows of no reason why it should not also be applicable in this affection.

Dr. Armstrong explained the production from cystitis of enlarged prostate as follows: In enlargement of the prostate there is always more or less congestion of the mucous membrane, which can be easily excited to inflammation. Again, there is some retention of urine; it becoming decomposed, acts as an irritant, and we have the most common cause of cystitis, He is not sure whether enlarged prostate is ever the result of cystitis, except as above indicated. He has used common salt numbers of times in washing out the bladder.

Dr. Calhoun inquired if the Academy had ever noticed an enlargement of the third lobe, or middle prostate gland. In such a case there was found an enlargement sufficient to produce obstruction in urinating, whilst the two lateral lobes were of normal size.

Dr. John M. Johnson reported the case of a lady confined 31st of last January, and delivered with forceps by Dr. Miller and himself. On the 12th day the right leg was attacked with milk-leg. The leg was elevated and continually rubbed upwards, but with no decided improvement. He then applied a tight roller bandage, which relieved the trouble. On the 19th day the left leg showed symptoms of the trouble. In a few hours the whole extent of the limb, including the buttock, became enormously swollen; toes strutting and extremely painful. When first attacked the lochia had ceased, but had been reinduced. They now ceased again. Rubbing having been again tried with no benefit, the bandage was tightly applied, from the toes upwards, embracing well the buttock. This relieved the pain in a few hours. At the same time he endeavored to relax the womb, and succeeded on the 32d day after delivery. He mentioned this case because it rather contravened his opinion, heretofore expressed, as to the proper treatment of milk-leg. It is now his opinion that if the swelling is confined to the calf, then rubbing suffices to remove the thrombus; but if it extends to the buttocks, then use the bandage.

Dr. John M. Johnson was called to see a lady, aged sixty years, who had been paralytic for ten or fifteen years—the right side having been immovable for nearly a year. She had previously had frequent apoplectic seizures, together with great nervousness; the whole having culminated at the time of his seeing her in a large carbuncle, on the back, near the last dorsal vertebra, from which a slough, about four by fourteen inches, had been thrown off. She at the same time complained of pain in the left leg, which was greatly swollen, and with all the other symptoms of milk-leg. The tight bandage was applied, and gave great relief; the limb being much reduced in size, and the pain less at the time of her death, which occurred one week after his first visit. He was unable to determine the cause of the milk-leg in this case.

The President corroborated the above statement of facts, and stated, in addition, that there was great sensibility from the groin along the course of the vessel; also, that there had been a continuous offensive discharge from the womb for a long time. He remarked further, in connection with the subject of milk-leg, that during the war he had a soldier under his charge whose leg was swollen, and he then gave it as his opinion that it was milk-leg, from obstruction of the circulation. Post mortem examination developed a complete closure of the femoral vessel, from pus, the result of a wound.

Whilst he believes that by friction we may be enabled to press water through the areolar tissue, and thus lessen the pain and swelling, he does not believe it would remove the obstruction. It is evident that no friction on the buttock could overcome the trouble, because the depth of the vessels would prevent sufficient force being exerted to remove the obstruction.

Dr. Armstrong called to mind two cases in his practice in which milk-leg came on independent of labor. In the first case a lady having had an attack of acute dysentery, was making good recovery, when both legs suddenly became affected with milk-leg. She recovered entirely. The other case was that of a lady with a fracture at the hip joint. Milk-leg supervened, the leg being immensely swollen. It finally yielded after some months, and she now used the leg with some facility.

Dr. John M. Johnson, from the great relief that followed the application of a tight bandage in his case, where the pain was confined principally to the course of the sciatic nerve, has been led to believe that in sciatica, by applying a bandage as tight as possible, and then a broad belt around the hips, and a pad in front, we would have a remedy of great efficiency.

Academy adjourned.

